# Prevalence and Factors Affecting the Optimal and Non-optimal Peak Inspiratory Flow Rate in Stable and Exacerbation Phases of Chronic Obstructive Pulmonary Disease and Bronchial Asthma in India

**DOI:** 10.7759/cureus.58670

**Published:** 2024-04-21

**Authors:** Selvaraj Murugaiya, Buvaneshwari Murugesan, Prasad S

**Affiliations:** 1 Department of Respiratory Medicine, Trichy SRM Medical College Hospital and Research Centre, Trichy, IND; 2 Department of Community Medicine, K.A.P. Viswanatham Government Medical College, Trichy, IND

**Keywords:** chronic obstructive pulmonary disease, bronchial asthma, spirometry, inhaler devices, peak inspiratory flow rate, copd exacerbations, bronchial asthma exacerbation

## Abstract

Introduction: Chronic obstructive pulmonary disease (COPD) and bronchial asthma pose significant threats and challenges to global health care, emphasizing the need for precise inhaler therapies to overcome this burden. The optimal peak inspiratory flow rate (PIFR) is a crucial determinant for the right selection and effective use of an inhaler device. It also helps to improve the treatment effectiveness of obstructive airway diseases worldwide as it allows effective drug delivery to distal airways and lung parenchyma. It is used as a selection criterion by physicians around the world for selecting personalized inhaler devices.

Objective: To find out the optimal and non-optimal PIFR prevalence and its influencing factors in stable and exacerbation phases of COPD and bronchial asthma in Tamil Nadu, India.

Methodology: It is a single-center, observational, cross-sectional study conducted from February 2022 to August 2023. The patients who meet the diagnostic criteria specified by the Global Initiative for Chronic Obstructive Lung Disease (GOLD) guidelines for COPD and the Global Initiative for Asthma (GINA) guidelines for bronchial asthma are enrolled in our study. The PIFR was measured using a hand-held digital spirometry device, along with demographic data collection. Statistical analyses, including t-tests and chi-square tests, were performed using SPSS version 21 (IBM Corp., Armonk, NY).

Results: Gender, height, and disease severity significantly impacted the PIFR. Females, normal BMI individuals, and those with moderate disease severity exhibited higher optimal PIFR rates. Stable or exacerbation phases, disease, and smoking status do not influence either optimal or non-optimal PIFR. Notably, substantial differences in lung function parameters were observed between optimal (60-90 L/min) and non-optimal PIFR (insufficient: <30 L/min, suboptimal: 30-60 L/min, excessive: >90 L/min) groups, highlighting their impact on respiratory health.

Conclusion: This study emphasizes the importance of personalized inhaler strategies, considering gender, height, and disease severity. Proper inhaler device selection, continuous monitoring of inhaler technique, and tailored inhaler education at every OPD visit are vital for optimizing effective COPD and bronchial asthma management and improving adherence to treatment.

## Introduction

Among all the known chronic respiratory disorders, chronic obstructive pulmonary disease (COPD) and bronchial asthma both contribute a major share of respiratory illnesses around the world, and some others are occupational lung diseases and pulmonary hypertension. These impose a substantial burden on global healthcare systems and individuals alike. The prevalence of COPD in India varies across different regions and populations. The prevalence of COPD in India was estimated to be around 7.0% in 2022 [1].

In India, between 1990 and 2016, the crude prevalence of COPD increased by 29%, while the crude prevalence of bronchial asthma climbed by 9% [2]. The prevalence of bronchial asthma in India will contribute 13.09% of the global burden in 2022. Both diseases are characterized by airflow limitation and are often associated with episodes of exacerbation, that lead to increased hospitalization, morbidity, mortality, disability-adjusted life years (DALYs), and diminished quality of life for the affected individuals and their families. Obstructive airway diseases are preventable diseases, despite the fact that COPD causes 8.7% of the total mortality in India and morbidity in 55.3 million people, contributing 4.8% of DALYs in the country, and bronchial asthma causes 13.2 thousand deaths and 27.9% of the DALYs in India [2].

Several definitions of an exacerbation of COPD and asthma have been put forth by various groups. An exacerbation of COPD is best defined as a prolonged worsening of the patient's condition, from the stable state and beyond normal day-to-day variations, which is acute in onset and may warrant additional treatment in a patient with underlying COPD [3]. The Global Initiative for Asthma (GINA) guidelines define “acute exacerbations” (asthma attacks or acute asthma) as “episodes of progressive increase in shortness of breath, cough, wheezing, or chest tightness, or some combination of these symptoms, accompanied by decreases in expiratory airflow that can be quantified by measurement of lung function” [4].

Assessing the pulmonary function is pivotal, as it helps to confirm the diagnosis, decide the management according to the severity of the conditions, and monitor the progress of the disease while the patient is on inhaler therapy. Despite inhaler therapy, in Indian patients with obstructive airway diseases (OAD), especially bronchial asthma, 0% are well controlled, 60% are partially controlled, and 40% have uncontrolled OAD [5]. The reasons for poor control are 35.9% poor inhaler device selection (OR: 3.65), 76.8% poor adherence (OR: 1.8), 17% poor inhaler technique (OR: 3.03), and poor buying capacity [6]. Poor inhaler device selection leads to poor adherence to inhaler therapy by patients. These two parameters together contribute significantly to the poor control of COPD and asthma in India.

Among all the parameters measured in a pulmonary function test, the peak inspiratory flow rate (PIFR) plays a pivotal role in selecting personalized inhaler devices, which measure the maximum flow rate attained during inhalation. Every inhaler device requires a different inspiratory flow rate and technique for proper drug delivery to the distal airways and lung parenchyma [7]. The PIFR was influenced by many factors, as per previous studies, including female gender, shorter height, older age, severity of disease, and a lower percent predicted forced vital capacity (FVC) and inspiratory capacity [8].

The measured PIFRs were classified into four categories: excessive PIFR (≥90 L/min), optimal PIFR (60-89 L/min), suboptimal PIFR (30-59 L/min), and insufficient PIFR (<30 L/min) [7-9]. By using the criteria of drug distribution and deposition, we categorize PIFR into optimal PIFR and non-optimal PIFR. Understanding the difference between optimal and non-optimal PIFR is essential while tailoring personalized inhaler therapies for patients. The optimal PIFR is 60-90 L/min; at this PIFR, the deagglomeration between active drugs and sugar carriers is sizable, and drug delivery to the peripheral airways and lung parenchyma happens effectively. The inadequate and suboptimal PIFR associated with poor deposition of inhaled drugs in the lung will result in unsatisfied efficacy and a potentially poor prognosis [10,11]. Compared to optimal PIFR, excessive PIFR also leads to more oropharyngeal deposition and less lung deposition [9,12]. The proportion of excessive, optimal, suboptimal, and insufficient PIFRs was 42%, 57%, 1%, and 0%, respectively, against low-resistance devices [13].

This study aims to bridge this critical knowledge gap by investigating the prevalence of optimal and non-optimal PIFR and identifying the factors that influence this in both the stable and exacerbation phases of COPD and bronchial asthma. By comprehensively examining these aspects, healthcare providers can gain valuable insights into the challenges faced by patients, enabling them to optimize inhaler therapies and improve disease management strategies. This research is not only pertinent for enhancing individual patient care but also holds the potential to inform broader healthcare policies, ultimately reducing the socioeconomic burden of COPD and bronchial asthma on both patients and healthcare systems.

## Materials and methods

Study design

We conducted a single-center, observational, cross-sectional study to determine the prevalence and factors affecting the optimal and non-optimal PIFR in stable and exacerbating COPD and bronchial asthma. We enrolled 313 patients in this study from February 2022 to August 2023, who were >14 years old, irrespective of gender, residence, or whether they had a history of breathing difficulty and met the diagnostic criteria of either COPD or asthma. All patients underwent spirometry measurements using hand-held digital spirometer equipment following the American Thoracic Society (ATS) performance standards [14]. Out of these, 152 patients were diagnosed with COPD, and 161 patients were diagnosed with bronchial asthma, meeting the diagnostic criteria established by the Global Initiative for Chronic Obstructive Lung Disease (GOLD) 2019 report or the Global Strategy for Asthma Management and Prevention (GINA 2019 update), respectively, including medical history, symptoms, and pulmonary function tests, considering stable and exacerbation phases of COPD and asthma separately [15,16].

For each patient to be enrolled in this study, an unambiguous pulmonary function test result that supported the diagnosis of COPD (forced expiratory volume in one second (FEV1)/FVC <70% after bronchodilator therapy without meeting the post-bronchodilator FEV1 reversibility criteria of 12% and 200 ml reversibility from pre-bronchodilator reversibility) and bronchial asthma (who is meeting the post-bronchodilator FEV1 reversibility criteria of 12% and 200 ml reversibility from pre-bronchodilator reversibility) was used.

In accordance with GINA and GOLD, an asthma exacerbation is characterized by a change in the patient's symptoms and lung function from the patient's normal state, and a COPD exacerbation is defined as an acute deterioration of respiratory symptoms requiring hospitalization. The low PIFR measured by spirometry was associated with suboptimal PIFR measured by using an In-Check DIAL device (Alliance Tech Medical, Granbury, TX) [17]; for this reason, we are taking direct hand-held digital spirometry PIFR measurements in our study instead of using an In-Check DIAL PIFR measurement.

Exclusion criteria

The exclusion criteria were as follows: (1) the patient refused to sign the informed consent; (2) the patient had interstitial lung disease or pulmonary embolism; (3) the patient had a cardiac failure, a neurological disorder, or a psychiatric disorder; (4) the patient was <14 years old.

Data analysis

The distribution of PIFR in all patients was described using descriptive statistics, as well as the demographic and clinical trials of the patients. Mean and standard deviation are used to describe continuous data, whereas frequency and percentage are used to describe categorical ones. Patients were categorized based on PIFR measurements: optimal (60-90 L/min) and non-optimal (PIFR <60 L/min and >90 L/min). An unpaired t-test was conducted to compare patient characteristics between these groups. Additionally, chi-square analysis was employed to compare categorical variables among the PIFR groups. A significance level of p ≤ 0.05 was set to determine statistical significance. The analysis was conducted using SPSS version 21 (IBM Corp., Armonk, NY).

Ethical considerations

This study adhered to ethical guidelines and obtained approval from the institutional review board. Patient confidentiality and data privacy were strictly maintained throughout the study.

## Results

Over an 18-month period from February 2022 to August 2023, 313 patients were studied, including 70.6% (n = 221) of outpatients and 29.4% (n = 92) of inpatients with a history of breathing difficulty and a provisional diagnosis of COPD or bronchial asthma who underwent a spirometry test. Out of these, 51.43% (n = 161) of patients were confirmed to have bronchial asthma, and 48.56% (n = 152) of patients were confirmed to have COPD.

Table 1 describes the socio-demographic variables of the study participants. A study reveals that among individuals aged less than 60 years, 68.9% (n = 111) have bronchial asthma, while 54.6% (n = 83) have COPD. For those aged over 60 years, 31.1% (n = 50) are diagnosed with bronchial asthma, and a higher percentage, 45.4% (n = 69), are diagnosed with COPD, and the association was found to be statistically significant (p-value = 0.009, OR: 1.84, 95% CI: 1.16-2.92). Regarding gender, in females, 39.8% (n = 64) have bronchial asthma, and 35.5% (n = 54) have COPD. In males, these figures were slightly higher, with 60.2% (n = 97) having bronchial asthma and 64.5% (n = 98) suffering from COPD. Looking at the BMI classification, the majority of individuals with both conditions fall into the normal weight category, i.e., 42.2% (n = 68) for bronchial asthma and 41.4% (n = 63) for COPD. Smokers are significantly (61.8%, n = 94) associated with COPD, and most of the bronchial asthma patients (63.4%, n = 102) are non-smokers, and the association was found to be statistically significant (p-value = 0.001, OR: 2.8, 95% CI: 1.7-4.4). Lastly, considering the status of the disease condition, 77% (n = 124) of bronchial asthmatics and 63.8% (n = 97) of COPD patients have stable disease conditions, while 36.2% (n = 55) of COPD patients and 22.9% (n = 37) of bronchial asthma patients have had exacerbations and hospitalizations, and the association was found to be statistically significant (p-value = 0.01, OR: 0.526, 95% CI: 0.321-0.863). This states that stable disease is more common in bronchial asthma patients, and exacerbation and hospitalization are more common in COPD patients. The very severe form of the disease (FEV1 <30%) was more common in COPD (13.2%, n = 20) compared with bronchial asthma patients (7.5%, n = 12).

**Table 1:** Sociodemographic variables compared with bronchial asthma and COPD patients. COPD: chronic obstructive pulmonary disease; OR: odds ratio; CI: confidence interval; BMI: body mass index; MRC: Medical Research Council Dyspnoea Scale; FEV1: forced expiratory volume in one second. Test used: Pearson chi-square. * P-value significance level <0.05.

S. No.	Variables	Category	Bronchial asthma, n (%)	COPD, n (%)	P-value	OR (95%CI)
1	Age category	≤60	111 (68.9)	83 (54.6)	0.009*	1.84 (1.16-2.92)
		>60	50 (31.1)	69 (45.4)		
2	Gender	Female	64 (39.8)	54 (35.5)	0.441	1.19 (0.75-1.89)
		Male	97 (60.2)	98 (64.5)		
3	BMI classification	Normal	68 (42.2)	63 (41.4)	0.983	-
		Obese	51 (31.7)	48 (31.6)		
		Underweight	42 (26.1)	41 (27.0)		
4	Smoking status	Non-smoker	102 (63.4)	58 (38.2)	0.001*	2.8 (1.77-4.43)
		Smoker	59 (36.6)	94 (61.8)		
5	Status of condition	Acute exacerbation	37 (23)	55 (36.2)	0.01*	0.52 (0.32-0.86)
		Stable	124 (77)	97 (63.8)		
6	MRC score	0.0	19 (11.8)	6 (3.9)		
		1.0	34 (21.1)	37 (24.3)	0.08*	-
		2.0	52 (32.3)	49 (32.2)		
		3.0	40 (24.8)	37 (24.3)		
		4.0	16 (9.9)	23 (15.1)		
7	Severity (FEV1)	<30%	12 (7.5)	20 (13.2)	0.34	-
		30-49%	66 (41)	62 (40.8)		
		50-80%	73 (45.3)	59 (38.8)		
		>80%	10 (6.2)	11 (7.2)		

Table 2 describes the data comparing height, weight, BMI, and various pulmonary function test (PFT) variables between two groups (bronchial asthma and COPD) and indicates the statistical significance. For height, weight, and BMI, there were no significant differences between the bronchial asthma and COPD groups. Among the PFT parameters, significant differences were observed in forced expiratory volume (FEF) 25-75% (p = 0.032, mean difference = -0.091) and FEF 25-75% percentage predicted (p = 0.012, mean difference = 3.61). Other parameters like FVC, FVC percentage predicted, FEV1, FEV1 percentage predicted, FEV1/FVC ratio, peak expiratory volume (PEF), forced expiratory technique (FET), forced inspiratory vital capacity (FIVC), and especially the PIFR did not show statistically significant differences between COPD and bronchial asthma.

**Table 2:** Pulmonary function test variables compared with bronchial asthma and COPD patients. COPD: chronic obstructive pulmonary disease; BMI: body mass index; FVC: forced vital capacity; FEV1: forced expiratory volume in one second; FEF: forced expiratory volume; PEF: peak expiratory volume; FET: forced expiratory technique; FIVC: forced inspiratory vital capacity; PIFR: peak inspiratory flow rate. Test used: t-test. * P-value significance level <0.05.

S. No.	Variables	Bronchial asthma (mean ± SD)	COPD (Mean ± SD)	p-value	Mean difference
1	Height	158.63 ± 9.61	158.19 ± 9.8	0.687	0.443
2	Weight	57.47 ± 12.7	56.53 ± 12.69	0.513	0.939
3	BMI	22.97 ± 5.23	22.56 ± 4.97	0.474	0.414
4	FVC	2 ± 0.73	1.88 ± 0.78	0.159	0.120
5	FVC predicted	70.44 ± 19.17	69.63 ± 23.65	0.738	0.813
6	FEV1	1.21 ± 0.52	1.11 ± 0.54	0.089	0.103
7	FEV1 predicted	52.03 ± 17	50.9 ± 20.05	0.588	1.13
8	FEV1/FVC	59.76 ± 8.27	58.21 ± 9.13	0.115	1.55
9	FEF 25-75%	0.68 ± 0.37	0.59 ± 0.39	0.032*	0.091
10	FEF 25-75% predicted	26.34 ± 13.01	22.72 ± 12.22	0.012*	3.61
11	PEF (L/S)	3.07 ± 1.25	2.89 ± 1.3	0.203	0.184
12	PEF (L/S) % predicted	46.48 ± 16.41	44.53 ± 17.84	0.316	1.945
13	FET (S)	6.32 ± 1.05	6.37 ± 1.26	0.684	-0.053
14	FIVC (L)	1.77 ± 0.8	1.6 ± 0.75	0.061	0.165
15	FIVC (L) % predicted	61.75 ± 22.42	58.91 ± 22.92	0.269	2.83
16	PIFR (L/min)	137.8 ± 60.93	132.45 ± 60.38	0.436	5.35

Table 3 illustrates the association between a patient’s anthropometric and clinical characteristics and their optimal and non-optimal PIFR. Male patients (65.17%, n = 174) are significantly associated with non-optimal PIFR compared to female patients (34.83%, n = 93, p = 0.012, OR = 2.22, 95% CI = 1.18-4.19). Disease severity, especially moderate severity (p = 0.001), is significantly associated with non-optimal PIFR. Compared to optimal PIFR (4.35), the most severe form of disease is more common in non-optimal PIFR (7.12), which is statistically significant. Less than 60 years old patients (63.7%, n = 170) have a higher non-optimal PIFR than >60 years old patients (36.3%, n = 97), but this is not statistically significant. Normal BMI (43.45%, n = 116) patients have a higher non-optimal PIFR than underweight (25.47%, n = 68) patients, but this is not statistically significant. Compared with smokers, non-smokers have a greater number of non-optimal PIFRs, but this is not statistically significant. Most of the non-optimal PIFR patients have stable disease. Most of the optimal and non-optimal PIFR patients have dyspnea levels score of 2, measured by the Medical Research Council Dyspnoea Scale (MMRC), but this did not significantly impact PIFR. In summary, male gender and moderate disease severity were key determinants of non-optimal PIFR variations among the patients studied, and this was statistically significant according to Pearson’s chi-square test (p < 0.05).

**Table 3:** Sociodemographic variables compared with compared with optimal and non-optimal PIFR category patients. PIFR: peak inspiratory flow rate; BMI: body mass index; MRC: Medical Research Council Dyspnoea Scale. Test used: Pearson chi-square. * P-value significance level <0.05.

S. No.	Variables	Category	PIFR Category		p-value	
			Optimal, n (%)	Non-optimal, n (%)		OR (95% CI)
1	Age category	≤60	24 (52.2)	170 (63.7)	0.14	0.622 (0.33-1.16)
		>60	22 (47.8)	97 (36.3)		
2	Gender	Female	25 (54.35)	93 (34.83)	0.012*	2.22 (1.18-4.19)
		Male	21 (45.65)	174 (65.17)		
3	BMI classification	Normal	15 (32.61)	116 (43.45)	0.363	-
		Obese	16 (34.78)	83 (31.09)		
		Underweight	15 (32.61)	68 (25.47)		
4	Smoking status	Smoker	25 (54.35)	128 (47.94)	0.43	0.774 (0.41-1.44)
		Non-smoker	21 (45.65)	139 (52.06)		
5	Status of condition	Acute exacerbation	9 (19.57)	83 (31.09)	0.113	0.539 (0.249-1.16)
		Stable	37 (80.43)	184 (68.91)		
6	MRC score	0.0	3 (6.52)	22 (8.24)	0.824	-
		1.0	8 (17.39)	63 (23.6)		
		2.0	17 (36.96)	84 (31.46)		
		3.0	11 (23.91)	66 (24.72)		
		4.0	7 (15.22)	32 (11.99)		
7	Severity	<30	8 (17.39)	24 (8.99)	0.001*	-
		30-49	28 (60.87)	100 (37.45)		
		50-80	8 (17.39)	124 (46.44)		
		>80	2 (4.35)	19 (7.12)		

In comparison with the optimal group, the non-optimal PIFR group of individuals had significantly higher demographic values in weight (53.37 vs. 57.64 kg, p = 0.035, mean difference = - 4.275) and height (153.54 vs. 159.6 cm, p = 0.001, mean difference = - 5.715), which is statistically significant. The BMI (p = 0.924) is not statistically significant. Except for FEV1/FVC ratio and FET (s), other pulmonary function parameters like FVC (1.42 vs. 2.03, p = 0.001), FEV1 (0.82 vs. 1.22), FEF 25-75% (0.43 vs. 0.67), PEF (2.07 vs. 3.14), FIVC (1.17 vs. 1.77), and PIFR (80.2 vs. 144.68) had statistically significant higher values in the non-optimal group of individuals (all p < 0.001). These results indicate substantial differences in lung function parameters between the optimal and non-optimal groups, underscoring the importance of these factors in respiratory health (Table 4).

**Table 4:** Pulmonary function test variables compared with optimal and non-optimal PIFR category patients. BMI: body mass index; FVC: forced vital capacity; FEV1: forced expiratory volume in one second; FEF: forced expiratory volume; PEF: peak expiratory volume; FET: forced expiratory technique; FIVC: forced inspiratory vital capacity; PIFR: peak inspiratory flow rate. Test used: t-test. * P-value significance level <0.05.

S. No.	Variables	PIFR category		p-value	
		Optimal, n (%)	Non-optimal, n (%)		Mean difference
1	Height	153.54 ± 11.63	159.26 ± 9.08	0.001*	-5.715
2	Weight	53.37 ± 15.37	57.64 ± 12.08	0.035	-4.275
3	BMI	22.84 ± 7.28	22.76 ± 4.64	0.924	0.078
4	FVC	1.42 ± 0.43	2.03 ± 0.76	0.001*	-0.613
5	FVC predicted	59.48 ± 17.78	71.87 ± 21.51	0.001*	-12.38
6	FEV1	0.82 ± 0.3	1.22 ± 0.55	0.001*	-0.398
7	FEV1 predicted	42.63 ± 17.43	53.01 ± 18.31	0.001*	-10.37
8	FEV1/FVC	57.64 ± 9.04	59.24 ± 8.66	0.249	-1.605
9	FEF 25-75%	0.43 ± 0.23	0.67 ± 0.39	0.001*	-0.236
10	FEF 25-75% predicted	19.63 ± 11.11	25.44 ± 12.82	0.004*	-5.808
11	PEF (L/S)	2.07 ± 0.64	3.14 ± 1.29	0.001*	-1.07
12	PEF (L/S) % predicted	35.61 ± 10.62	47.24 ± 17.46	0.001*	-11.63
13	FET (S)	6.25 ± 1.26	6.36 ± 1.13	0.539	-0.113
14	FIVC (L)	1.17 ± 0.45	1.77 ± 0.79	0.001*	-0.601
15	FIVC (L) % predicted	48.11 ± 15.92	62.49 ± 23.02	0.001*	-14.37
16	PIFR (L/min)	80.2 ± 7.42	144.68 ± 60.74	0.001*	-64.47

Table 5 shows that the binary logistic regression analysis revealed that FVC, forced expiratory volume (FEV1% predicted), and FIVC (L) were significantly associated with the PIFR optimal and non-optimal categories. The PIFR category was significantly more strongly associated with FVC values (adjusted odds ratio (AOR): 23.74, 95% CI: 4.77-118.02), FEV1 % predicted (AOR: 0.968, 95% CI: 0.941-0.997), and FIVC (L) values (AOR: 0.074, 95% CI: 0.017-0.326).

**Table 5:** Binomial logistic regression CI: confidence interval; FVC: forced vital capacity; FEV1: forced expiratory volume in one second; FIVC: forced inspiratory vital capacity. Test used: binomial logistic regression. Model summary: Cox and Snell R2 for the model: 0.235. * P-value significance level <0.05.

Variables	Adjusted odds ratio	95% CI		p-value
		Lower	Upper	
FVC	23.747	4.778	118.029	0.001*
FEV1 % predicted	0.968	0.941	0.997	0.028*
FIVC	0.074	0.017	0.326	0.001*

## Discussion

In our study, we aim to find out the prevalence and the factors that influence optimal and non-optimal PIFR in both stable and exacerbation phases of COPD and bronchial asthma. Inhaler devices are commonly used for delivering inhaler medications worldwide, and each inhaler device possesses internal resistance for successful drug delivery. When choosing an inhaler device as part of a treatment plan for COPD or asthma, PIFR is used to evaluate a patient's capacity to generate enough inspiratory flow.

When patients exhibit either diminished PIFR (sub-optimal PIFR and insufficient PIFR) with an inspiratory flow rate of <60 L/min or excessive PIFR with an inspiratory flow rate of >90 L/min, they might struggle to get symptomatic relief because of ineffective or excessive inhalation, which limits drug deagglomeration and deposition into the peripheral airways and lung parenchyma and increases drug deposition in the oropharynx, which leads to poor disease control. Therefore, PIFR assessments are pivotal in ensuring the presence of optimal PIFR, which helps individualize patient-tailored inhaler device selection and treatment. In India, out of all available inhaler devices, the most commonly used inhaler device is the dry powder inhaler device due to its cost-effectiveness and ease of carrying in hand. These are mostly low-resistance devices with effective distal airway drug delivery [18].

In our study, the prevalence of excessive, optimal, suboptimal, and insufficient PIFR measured by a hand-held digital spirometer for bronchial asthma was 80.7%, 9.3%, 6.2%, and 3.7%, respectively; for COPD, it was 73.7%, 18.4%, 6.6%, and 1.3%, respectively. The proportion of excessive, optimal, suboptimal, and insufficient PIFRs was 42%, 57%, 1%, and 0%, respectively, against low-resistance devices [11]. Both studies confirm that the prevalence of excessive PIFR is high. In comparison with Chen et al.'s study, our study shows the prevalence of excessive PIFR, suboptimal PIFR, and insufficient PIFR was high, but optimal PIFR was low [13]. In our study, the prevalence of optimal PIFR (60-90 L/min) among bronchial asthma and COPD groups was 9.3% and 18.4%, and the suboptimal PIFR (<60 L/min) was 9.9% and 7.9%, respectively, whereas a study conducted by Sharma and colleagues in the outpatient setting of patients with advanced COPD found the prevalence of PIFR less than 60 L/min to be 19% [19]. In comparison with Sharma and colleagues, our study shows the prevalence of suboptimal PIFR is low. In this study, the prevalence of optimal PIFR (60-90 L/min) among bronchial asthma and COPD groups was 9.3% and 18.4%, and the prevalence of non-optimal PIFR (>90 L/min, 30-60 L/min, and <30 L/min) was 90.6% and 81.6%, respectively. This excessive PIFR contributes to the major junk of the prevalence of non-optimal PIFR (<60 L/min and >90 L/min) compared with sub-optimal and insufficient PIFR together in both COPD and bronchial asthma, causing wide variation in PIFR among the patients. This will mislead the physician in the selection of an inhaler device, as the optimal PIFR prevalence was very low compared with non-optimal PIFR (both bronchial asthma and COPD). This is one of the main reasons for the poor selection of personalized inhaler devices and the poor control of obstructive airway diseases, despite inhaler therapy. Our study supports Gold et al.'s (2014) study on the reasons for well-controlled (0%), partially controlled (60%), and uncontrolled (40%) obstructive airway diseases in India [5].

The male gender was associated with non-optimal PIFR compared with the female gender, which is statistically significant and inconsistent with previous studies, as those studies were taken between optimal PIFR and sub-optimal PIFR [20]. We also noted that <60-year-old patients tend to be linked with lower optimal PIFR, and this is not consistent with previous research. While our analysis did not show statistical significance between age and PIFR, younger age is associated with a lower optimal PIFR and is not consistent with other studies [17].

In the comparison with the optimal group, the non-optimal PIFR group of individuals had significantly higher demographic values in weight (53.37 vs. 57.64 kg, p = 0.035) and height (153.54 vs. 159.6 cm, p = 0.001), which is statistically significant. The correlation between BMI and PIFR in our study is not statistically significant, highlighting the insignificance of considering BMI while tailoring inhaler therapies [21]. The results from our study show a statistically significant correlation between the severity of obstruction (FEV1%) and PIFR; the very severe (FEV1 <30%), moderate (FEV1 50-80%), and mild (FEV1 >80%) forms of obstructive airway disease were more commonly associated with poorer optimal PIFR (17.39%, 17.39%, and 35%) compared with the severe (FEV1 30-49%) form of obstructive airway disease. The observation that the severity of airway obstruction is not consistently associated with non-optimal PIFR is consistent with previous studies. Comparing our findings with the research conducted by Prime et al. reveals that patients with milder disease severity consistently exhibited higher optimal PIFR rates [22]. This underlines the significance of disease severity as a determinant factor in inhaler effectiveness. Optimizing inhaler therapies for patients with varying disease severity levels could substantially improve treatment outcomes.

We also found that the influence of very severe and severe forms of COPD and bronchial asthma on PIFR might differ from its impact on mild and moderate forms of COPD and bronchial asthma. This complexity underscores that lung function and PIFR are affected by various factors beyond the degree of airway obstruction. Understanding these nuances is essential for tailoring inhaler therapies effectively. Comparing our findings with other studies, patients with very severe and severe disease severity consistently showed higher optimal PIFR rates, emphasizing the significance of disease severity in inhaler effectiveness. This underlines the importance of customizing inhaler technologies to accommodate patients' diverse needs, considering factors like gender, weight, height, and disease severity. Even though there are distinct factors affecting PIFR, our study revealed that stable and acute exacerbation phases have little impact on either optimal PIFR or non-optimal PIFR.

These findings emphasize the need for personalized inhaler strategies tailored to individual patient profiles. Healthcare providers should consider these identified factors while prescribing inhaler devices and teaching inhaler techniques, ensuring the patient has good adherence to the treatment that ensures optimal drug delivery and enhances treatment efficacy for patients with COPD and bronchial asthma. Further prospective studies are warranted to validate these findings and explore potential interventions to address the PIFR as a sole tool or use multivariable as a tool for customary inhaler device selection for good control of COPD and bronchial asthma.

The limitations of this study were that we cannot generalize these findings to a general population because the study was done in only one tertiary care center, and it is a cross-sectional study, so we cannot draw conclusions on the causality of associations between different factors. Using the PIFR instrument for measuring all the patients leads to instrumental and human error.

## Conclusions

This prospective analysis provides valuable insights into optimal and non-optimal PIFR instead of optimal and suboptimal PIFR, which were taken as subjects of study in older studies. Despite limitations, the study sheds light on the challenges faced by physicians in selecting inhaler devices for personalized inhaler therapies, as the optimal PIFR proportion is very low compared to non-optimal PIFR in our study, as it is one of the leading major causes of poor control in COPD and asthma patients, emphasizing the need for upgrading the device selection criteria along with PIFR for better, personalized inhaler therapies and continuous monitoring of inhaler technique for optimal disease management. To overcome this issue and gain a better understanding of factors influencing PIFR, we need more studies about optimal PIFR vs. non-optimal PIFR and sub-optimal vs. excessive PIFR, as they are important criteria for inhaler device selection.
